# Analysis of sulphadoxine/pyrimethamine resistance-conferring mutations of *Plasmodium falciparum *from Mozambique reveals the absence of the dihydrofolate reductase 164L mutant

**DOI:** 10.1186/1475-2875-6-35

**Published:** 2007-03-23

**Authors:** Natércia Fernandes, Paula Figueiredo, Virgilio E do Rosário, Pedro Cravo

**Affiliations:** 1 Departamento de Pediatria, Universidade Eduardo Mondlane/Faculdade de Medicina. Av. Salvador Allende, CP 257, Maputo, Mozambique; 2 UEI Malária, Centro de Malária e Outras Doenças Tropicais/IHMT, Universidade Nova de Lisboa, Rua da Junqueira, 96 1349-008, Lisbon, Portugal; 3 UEI Biologia Molecular, Centro de Malária e Outras Doenças Tropicais/IHMT, Universidade Nova de Lisboa, Rua da Junqueira, 96 1349-008, Lisbon, Portugal

## Abstract

**Background:**

*Plasmodium falciparum *is the predominant human malaria species in Mozambique and a lead cause of mortality among children and pregnant women nationwide. Sulphadoxine/pyrimethamine (S/P) is used as first line antimalarial treatment as a partner drug in combination with artesunate.

**Methods:**

A total of 92 *P. falciparum*-infected blood samples, from children with uncomplicated malaria attending the Centro de Saude de Bagamoyo in the Province of Maputo-Mozambique, were screened for S/P resistance-conferring mutations in the *pfdhfr *and *pfdhps *genes using a nested mutation-specific polymerase chain reaction and restriction digestion (PCR-RFLP). The panel of genetic polymorphisms analysed included the *pfdhfr *164L mutation, previously reported to be absent or rare in Africa.

**Results:**

The frequency of the S/P resistance-associated *pfdhfr *triple mutants (51I/59R/108N) and of *pfdhfr/pfdhps *quintuple mutants (51I/59R/108N *+ *437G/540E) was 93% and 47%, respectively. However, no *pfdhfr *164L mutants were detected.

**Conclusion:**

The observation that a considerably high percentage of *P. falciparum *parasites contained S/P resistance-associated mutations raises concerns about the validity of this drug as first-choice treatment in Mozambique. On the other hand, no *pfdhfr *164L mutant was disclosed, corroborating the view that that this allele is still rare in Africa.

## Background

Mozambique is included in the top ten nations most affected by malaria, with *Plasmodium falciparum *being the predominant species. Malaria is of stable transmission and endemic in the entire country, making it the major cause of morbidity and mortality across all age groups, accounting for a third of all hospital deaths (USAID Mozambique Country Strategic Plan, FY 2004–2010). The choice of appropriate therapeutic policies that circumvent parasite chemoresistance has been one of the major challenges for malaria control nationwide. As the intensity of chloroquine resistance increased, the country implemented a change of first-line antimalarial treatment in 2002 to a combination of sulphadoxine/pyrimethamine (S/P) + amodiaquine. In 2004, this has been further altered to S/P + artesunate, in line with current WHO recommendations for the use of Artemisinin Combination Therapies (ACTs) [1].

Sulphadoxine and pyrimethamine (S/P) act as synergistic inhibitors of folate biosynthesis which, in malaria parasites, is an obligatory requirement for the production of nucleotides and hence DNA synthesis. Because both compounds act synergistically, any loss of efficiency in either component results in the reduction of the effectiveness of the combination as whole. In this context, the occurrence of certain molecular polymorphisms in the dihydrofolate reductase (*pfdhfr*) and dihydropteroate synthase (*pfdhps*) genes have been associated to *in vivo *S/P treatment outcome [2,3]. Particularly in East Africa, the occurrence of the so-called *pfdhfr/pfdhps *quintuple mutant parasites (*dhfr *51I/59R/108N *+ dhps *437G/540E) appears to be a good predictor of S/P treatment failure [4,5].

Previous studies in Asia and South America have demonstrated that *pfdhfr *mutants harboring a change from I to L at position 164 in concert with 108N plus 51I and/or 59R mutations present high-level resistance to both pyrimethamine and cycloguanil [6,7]. It has been suggested that this quadruple mutant has been selected through continued use of S/P in natural parasite populations [8-11]. Fortunately, *P. falciparum *I164L mutants have not consistently been detected in Africa so far [12]. Nevertheless, because S/P is safe and cost-effective, its use in Africa is growing either as first-line treatment alone or as a partner drug within a combination, increasing the likelihood of selection of I164L mutants. In face of such a scenario, routine molecular surveillance will be needed to detect the eventual emergence and propagation of this mutation.

The present work reports the prevalence of mutations in codons 51, 59, 108 and 164 of the *pfdhfr *gene and in codons 437 and 540 of the *pfdhps *gene among *P. falciparum *samples collected from symptomatic malaria-infected children from Maputo, Mozambique.

## Methods

The study was conducted from July to October 2004, among symptomatic children attending in Bagamoyo Health Centre in Maputo City, Mozambique. One hundred children with uncomplicated malaria were enrolled. Inclusion criteria were i) monoinfection with *P. falciparum*, ii) no intake of antimalarial drugs during the last three weeks, iii) no signs of complications (patients who developed signs of complications were immediately transferred for adequate treatment and excluded from the study), iv) no history of allergic reactions to sulphonamides, and v) informed consent of a parent or guardian. The study was ethically reviewed and approved by the "Comité Nacional de Bioética para a Saúde", the local Ethics Committee. At each visit of the patients to the centre, thin and thick blood films were prepared from finger-pricked blood and 50 μl of blood were dotted on Whatman (Maidstone, United Kingdom) 3 MM filter paper and air-dried at room temperature. The presence of *P. falciparum *in the peripheral blood was determined by microscopical examination of thick blood films in slides stained with 5% Giemsa solution. Parasite quantification was done by calculating the percentage of *P. falciparum*-infected red blood cells upon examination of thin blood films prepared as above.

The preparation of parasite genomic DNA, nested mutation-specific PCR and detection of point mutations at *pfdhfr *(codons 51, 59, 108 and 164) and *pfdhps *(codons 437 and 540) was done using previously published protocols [13]. The frequency of mutant, wild-type and mixed genotypes was assessed for each individual polymorphic marker. To calculate the frequency of parasites carrying multiple mutations, mixed genotypes were excluded in order to maximize the probability that the observations reflected genuine haplotypes and not a fictitious combination of mutations from different clones.

## Results and discussion

Of a total one hundred children recruited into this study, ninety two satisfied the inclusion criteria. The median age of the 92 patients (50 female, 42 male) was 7.3 years (range 3 months to 15 years). The geometric mean asexual parasitaemia of *P. falciparum *was 2.33% (95% confidence interval, 0.50–5.68).

**Figure 1:**
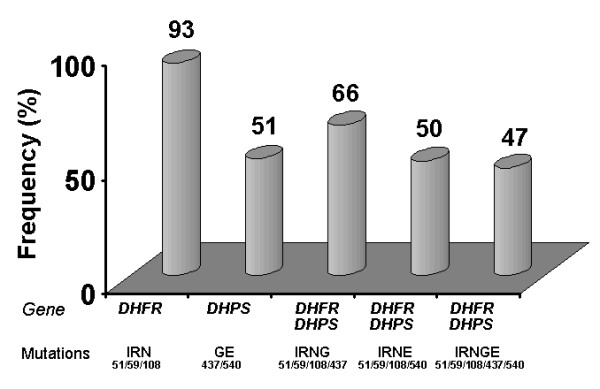
Frequencies of *Plasmodium falciparum *parasites from Maputo-Mozambique harboring more than one mutation in the *pfdhfr *and/or *pfdhps *genes.

The main observations resulting from the genotype analyses are presented as allele frequencies and haplotype frequencies in Table 1 and Figure 1, respectively. The frequency of mutations unveiled for both genes was high: 81 out of 92 isolates harbored the *dhfr *51I mutation, 82 of 90 were *dhps *59R mutants and 87 out of 92 displayed the 108N mutation (Table 1). *Dhps *mutants were less frequent than *dhfr *ones, but already well established among the parasite population (Table 1). Additionally, the proportion of parasites of parasites carrying more than one mutation at one or both genes (multiple mutants) proved to be significantly elevated (Figure 1). In this respect, attention is drawn to the observations that most of the parasite population carried triple *pfdhfr *mutations (*pfdhfr *51I/59R/108N) and that approximately half harbored the quintuple mutant genotype *pfdhfr *51I/59R/108N + *pfdhfr *437G/540E (Figure 1) that have been shown to predict S/P treatment failure in East Africa [4,5]. Indeed, in Mozambique, a previous study reported that two mutations at codon 59 in *dhfr *and codon 437 of *dhps *were actually enough to significantly predict parasitological failure [14]. Although in the present work no *in vivo *drug efficacy screening was carried out, the above genotype data appears to be consistent with previously reported S/P treatment failure for this region [15].

**Table 1:** Frequency of mutant, wild-type and mixed genotypes among the *pfdhfr *and *pfdhps *genes of *Plasmodium falciparum *from Maputo, Mozambique

	**GENE**					
	***Pfdhfr***				***Pfdhps***	
	
Codon	**51**	**59**	**108**	**164**	**437**	**540**
Mutant (%)	88	91	95	0	53	40
Wild-type (%)	7	6	2	100	35	41
Mixed (%)	5	3	3	0	12	19

In a recent survey in Malawi, the *pfdhfr *164L mutation, responsible for high level S/P resistance was reported [16]. In another study, which made use of a yeast expression system allowing detection of low levels DHFR-TS alleles, the presence of such mutants was also shown to occur in Tanzania [17]. These reports may be interpreted as early warning signs for a potential introduction of this genotype in Africa and prompted us to survey Mozambican parasites for this mutation. *Pfdhfr *164L mutants were not identified in the present investigation however (Table 1), corroborating previous findings indicating that this mutation has not yet established itself in Africa [18,19]. Although the number of isolates analysed in the current report may not be representative of the whole parasite population in this area, numerous other mutant alleles of *pfdhfr *and *pfdhps*, normally present in the region, were observed (Table 1). One of the possible explanations for the reported absence of this mutant could be that the 164L mutants may exist, but have not been detected using the standard PCR-RFLP protocol. Alternative explanations for the overall lack of this mutation among African parasites in general are put forward by Nzila and co-workers [12]. Their main premise is that the 164L mutation comes with a considerable fitness cost for the parasite and that the genetic composition of African parasites is less able to sustain it than their Asian counterparts. Nonetheless regular surveillance for this mutation should continue, in order to curtail the impact of S/P resistance spread in the African continent.

## Conclusion

The observed high frequency of mutations in the S/P resistance-associated *pfdhfr *and *pfdhps *genes raises evidence-based concerns about the use of this drug as a component of first-line antimalarial treatment in Mozambique. The continued deployment of S/P will increase drug-based selective pressure which progressively raises the proportion of drug-resistant mutants and leaves combination therapy highly dependant on the sole effect of the S/P partner drug.

The study of the mechanisms associated with the selection of 164L mutation and continuous monitoring of this and other mutations are required, especially in areas where S/P is extensively used as first-choice antimalarial treatment, as in Maputo-Mozambique.

## Authors' contributions

NF and PF carried out most of the experimental procedures and contributed for the elaboration of the manuscript. VEdR and PC conceived the study, participated in its design and co-ordination and were involved in phases of the experimental work.
